# Carnivores as Important Reservoirs of Intestinal Helminthic Infections in Mazandaran Province, Northern Iran

**Published:** 2018

**Authors:** Afsaneh AMOUEI, Hefzallah JAHANDAR, Ahmad DARYANI, Mehdi SHARIF, Shahabeddin SARVI, Azadeh MIZANI, Seyed Abdollah HOSSEINI, Mohammad SARAFRAZI, Abolghasem SIYADATPANAH, Shaban GOHARDIEH, Reza BASTANI, Shirzad GHOLAMI

**Affiliations:** 1. Toxoplasmosis Research Center, Mazandaran University of Medical Sciences, Sari, Iran; 2. Student Research Committee, Mazandaran University of Medical Sciences, Sari, Iran; 3. Mazandaran Provincial Veterinary Department of Medical Sciences, Sari, Iran; 4. Dept. of Parasitology, Faculty of Medicine, Mazandaran University of Medical Sciences, Sari, Iran; 5. Molecular and Cell Biology Research Center, Dept. of Parasitology, Mazandaran University of Medical Sciences, Sari, Iran

**Keywords:** Carnivores, Reservoirs, Intestinal helminthic, Iran

## Abstract

**Background:**

Intestinal parasites are the most common causes of gastrointestinal disease in canine. Stray dogs and wild candies can represent potential reservoirs of enteropathogens to other hosts. Therefore, present study determined the prevalence of intestinal parasites in canine in Mazandaran Province, northern, Iran.

**Methods:**

Overall, 58 small intestinal samples of animals (42 stray dogs and 16 jackals) were collected from Oct 2012 to Dec 2013. The intestine contents were studied to detect and identify helminth infections. Then, the helminths were collected and their morphological traits were identified.

**Results:**

Overall among infected stray dogs and jackals, 11 species were found. Three species of nematodes, seven species of cestodes and one trematode were observed. The prevalence of gastrointestinal helminths of stray dogs and jackals were 59.5% and 50%, respectively. Among registered zoonotic helminths *A. caninum* was the predominant parasite both stray dogs and jackals. Interestingly, *Spirometra* spp. was reported in these animals. Moreover, *A. caninum* showed a higher percentage rate in center region of province.

**Conclusion:**

There are the clear risks of zoonotic helminths parasites infection in this region. Therefore, understanding the epidemiology of zoonotic parasite infection is useful for health care access both domestic animals and humans health.

## Introduction

Intestinal parasites (protozoa and helminths) are the most common causes of gastrointestinal disease in canine. From the veterinary and medical points of view, stray dogs and wild candies can represent potential reservoirs of enteropathogens to human and domestic animals (1–3). A variety of canine parasites can be found in the gastrointestinal tract of dog and another canine. Among them, *Toxocara canis*, *Echinococus granulosus*, *Ancylostoma* spp., *Giardia* spp. and *Cryptosporidium* spp. have received great attention due to their risk and zoonotic implications especially to developing countries (4). Numerous epidemiologic surveys of intestinal parasites have been performed in different areas of the world. However, according to an estimated more than 60 zoonotic diseases are concerned with canine especially in dog, Jackal, and Fox, diverse factors can influence the frequency of infections in a community (5).

In Iran, searches have shown the different percentages of intestinal parasites (6–8). These studies indicated important information on parasitic infections at a local scale. Recently, intestinal parasite in canine in the North of Iran is reported (9). Due to special geographical conditions and close contact between human and these animals, it is important to identify some of the parasites and the risk of zoonotic transmission in the Caspian areas, north of Iran. Thus, in the endemic areas for determining the status of the major parasitic infections is need to study in every 5 to 10 yr.

Therefore, the present study aimed to determine the prevalence of intestinal parasites in canine. This research was designed to understanding the epidemiology of these parasitic infections, which are significant subjects for public health and animal health especially for developing new strategies for treatment and control of zoonotic disease in north of Iran.

## Materials and Methods

### Study area

This study was carried out in different areas in the Mazandaran Province, northern Iran. Mazandaran is divided into three parts plains, coastal and mountainous areas. This province covers an area of 23756 km and has a population over 3 million people. The climate is temperature humid with an annual rainfall of 500 mm and an average temperature of 17 ºC.

Overall, 58 fecal samples were collected from the stray dogs in Oct 2012 to Dec 2013. The animal’s population checked consisted of 42 stray dogs (*Canis familiaris)* and 16 jackals (*Canis aureus*). A number of stray dogs were assembled based on the stray dog control and prevention programs and others of them and the jackals were obtained by chance of road accident.

Animals were transported to the Parasitology Laboratory in Faculty of Medical School. The main characters of these animals (sex, approximate age, and area) were recorded. After autopsy, gastrointestinal tract in these animals was removed and opened entirely. The intestine contents were destined to detect and identify helminth infections.

Five grams of fecal specimens were taken from the rectum and were examined using Telemann sedimentation and also the formalin-ether concentration methods. Remaining contents were washed through 60 and 80 mesh wire series under tap water and the helminths were collected. The collecting parasites were preserved in warm 10% acid-formalin. Followed by cleared and stained (Nematodes in lacto phenol-tapeworms in acid alum carmine) their morphological traits were identified (9).

### Data analysis

Overall, the obtained data were analyzed using SPSS version 15 software (Chicago, IL, USA). The comparisons of prevalence’s between host gender and age were performed to evaluate difference in frequency of the helminth parasites using Chi-square test (*P*<0.05).

### Ethical issues

This project was approved by the relevant Ethics Committee in of Mazandaran University of Medical Sciences.

## Results

**Fig. 1:**
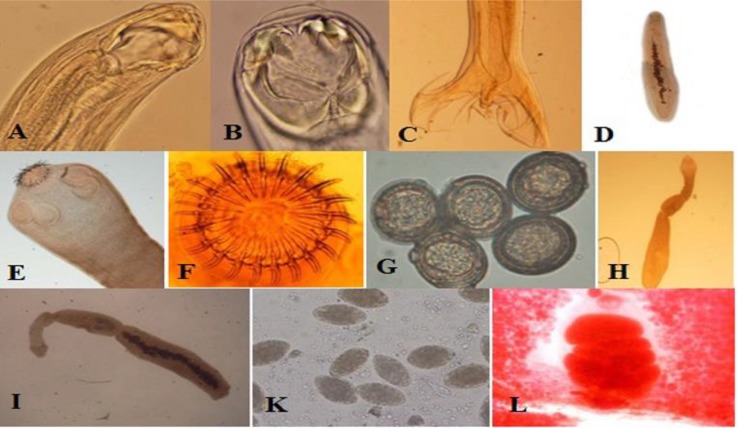
Figures of the gastrointestinal helminths were identified in stray dogs and jackals. A: *U. stenocephala* from dog, B: *A. caninum* from dog, C: Lateral view of male bursa of *A. caninum* from dog, D: *Barachylamia spp.* from dog X4, E: Scolex of *T. hydatigena* from dog, F: Rostellum of *T. hydatigena*, G: Egg of *Teania* spp. X40 from a dog, H: *E. granulosus* from jackal, I: *E. granulosus* from dog, k: Egg of *Spirometra spp.* X10 and L: proglottid of *Spirometra* spp. from dog and jackal (Original figures)

Among infected stray dogs and jackals, 11 species were found. Three species of nematodes; *T. canis, A. caninum, U. stenocephala*, seven species cestodes; *E. granulosus, T. hydatigena, T. multiceps, T. ovis, T. taenia formis, Spirometra* spp., *Mesocestoides* spp*., D. caninum, D. acanthotetra* and one trematode; *Barachylamia* spp. were observed (Fig. 1) (Table 1). *A. caninum* (35.71%), followed by *T. hydatigena* (30.95%) were the most prevalence in the stray dogs. In the jackals, the predominant species were *A. caninum* (56.25%) and *U. stenocephala, T. hydatigena* and *Mesocestoides* spp. (31.25%) (Table 1). Significant different was not found the influence of the region in prevalence of intestinal parasites, but only in the infection of *A. caninum* which a statistically higher prevalence in center parts both stray dogs and jackals (Table 2).

**Table 1:** Prevalence of helminth parasites from 42 stray dogs and 16 jackals in Northern Iran

***Parasites***	***No. of infected stray dogs***	***%***	***No. of infected jackals***	***%***
Nematoda				
*Toxocara canis*	4	9.52	2	12.5
*Ancylostoma caninum*	15	35.71	9	56.25
*Uncinaria stenocephala*	12	26.57	5	31.25
Cestoda				
*Echinicoccus granulosus*	12	28.57	3	18.75
*Taenia molticeps*	2	4.76	2	12.5
*Taenia hydatigena*	13	30.95	5	31.25
*Taenia ovis*	2	4.76	2	12.5
*Taenia taenia formis*	3	7.14	2	12.5
*Spirometra* spp.	1	2.38	1	6.25
*Mesocestoides* spp.	6	14.28	5	31.25
*Dipylidium caninum*	8	19.04	2	12.5
*Dipylidium acanthotetra*	-	-	1	6.25
Trematoda				
*Barachylamia spp.*	1	2.38	-	-

**Table 2:** Prevalence of helminth parasites from 42 stray dogs and 16 jackals by region in Northern Iran

***Region and canines Parasites***	***East***		***Center***		***West***	
	***Dog***	***Jackal***	***Dog***	***Jackal***	***Dog***	***Jackal***
	* n^a^ (%)	** n^b^(%)	**n^a^(%)**	**n^b^(%)**	**n^a^(%)**	**n^b^(%)**
Nematoda						
*Toxocara canis*	1 (2.38)	1 (6.25)	1 (2.38)	1 (6.25)	2 (4.76)	-
*Ancylostoma caninum*	5 (11.9)	-	10 (23.8)	6 (37.5)	8 (19.04)	3 (18.75)
*Uncinaria stenocephala*	3 (7.14)	-	5 (11.9)	4 (25)	4 (9.25)	1 (6.25)
Cestoda						
*Echinicoccus granulosus*	3 (7.14)	1 (6.25)	1 (2.38)	2 (12.5)	4 (9.52)	-
*Taenia molticeps*	-	-	1 (2.38)	1 (6.25)	1 (2.38)	-
*Taenia hydatigena*	1 (2.38)	-	3 (7.14)	1 (6.25)	1 (2.38)	-
*Taenia ovis*	2 (4.76)	-	2 (4.76)	-	-	-
*Taenia taenia formis*	2 (4.76)	-	2 (4.76)	-	1 (2.38)	-
*Spirometra* spp.	-	-	1 (2.38)	-	-	1 (6.25)
*Mesocestoides* spp.	3 (7.14)	-	4 (9.52)	-	4 (9.52)	-
*Dipylidium caninum*	2 (4.76)	-	4 (9.52)	2 (12.5)	2 (4.76)	-
*Dipylidium acanthotetra*	-	-	-	1 (6.25)	-	-
Trematoda				-	-	-
*Barachylamia*s pp.	-	-	1 (2.38)			

*n^a^: Number of infected Stray dogs

**n^b^: Number of infected jackals

## Discussion

In the current study, the overall prevalence of gastrointestinal helminths of stray dogs and jackals was 59.5% and 50%, respectively. Most of the parasite species identified in our work have a worldwide distribution (10–12). Although, comparing infection prevalence of intestinal helminths parasites in different studies could be sometimes difficult because there is diversity of demographic aspects in the population of these parasites. There have been some previous reports at different decades on the prevalence of parasites of stray dogs and jackals in the Mazandaran Province, north of Iran (8, 9).

The population of wild Canids cannot be controlled by the health system. Their free roaming and also their habits were exposed to more natural infection. This could be an important source of infection for humans and domestic animals (6). The present survey could also detect some of the zoonotic helminths such as *T. canis, A. caninum, U. stenocephala, D. caninum, Spirometra* spp., *E. granulosus and T. multiceps* in the north of Iran. This finding is important in medical, veterinary and public health in Iran and Eastern Mediterranean region, where animal husbandry and agriculture are the most important jobs. In these areas, dogs and jackals are the most important reservoirs for the transmission of infectious agents especially helminths parasites to animals and human.

In our study, among zoonotic helminths, *A. caninum* was the predominant parasite both stray dogs (35.71%) and jackals (56.25%) which has lower prevalence than previous studies (8, 9). In studied region, there were risks to public health of Cutaneous Larva Migrant (CLM) syndromes. In other countries, the reported infection rate was varied from 6% to 88% in the population (13, 14). *Toxocara canis* was detected as another zoonotic important parasite in this research.

The prevalence of toxocariasis in dog was almost increased, that related to sample small size in our study with only 12 jackals. In contrast with our work, a higher frequency rate of this nematode was reported in stray dogs (9), because all of samples in our work were adult animals. Older dogs may develop immunity whereas in pups could be infected transplacentally and transmammary that probably be incidence more susceptible for this nematode. Moreover, the high prevalence was recorded as visceral and ocular larva migrants in the world (2, 15, 16).

In this study, another zoonotic parasite that is serious threat to public health was *E. granulosus* whit high dissemination 28.57% in stray dogs and 18.75% in jackals.

Based on our knowledge, it may be related to the sylvatic cycle of parasite in this province. *E. granulosus* in the urban stray dogs was not reported, but another study in rural and forest area prevalence of parasite has shown 22.2% in stray dogs and (46.7%) of jackals. However, cosmopolitan prevalence of this helminth was reported from 1% to 63.5% from Iran [6]. In human, about 132 cases of cystic surgeries were performed during seven years from 2001 to 2007 in this area (17). Several intestinal helminths infections of dogs and jackals of Iran such as *E. granulosus*, *T. canis*, *U. stenocephala, D. caninum*, *D. immitis*, are revealed as important zoonotic diseases (6–9). Echinococcosis/hydatidosisis is considered as the most problematical helminthic infection for both public health and domestic animals in Iran. The prevalence of *E. granulosus* in dogs (46.7%) and jackals (22.2%) was reported in Mazandaran Province, north of Iran (8), but is in contrast in Iran (6), who could not find it in jackals. The presence of *E. granulosus* in in dogs, jackals and hydatid cyst in sheep, goats confirms the domestic cycle of hydatidosis in Iran (6, 7, 18). The relation of this cycle to the sylvatic cycle in different regions of Iran requires further research.

The common tapeworm, *D. caninum* was encountered just with low prevalence in comparison with other surveys. Prevalence of *D. caninum* in the current study was in accordance with previous research in jackals (6, 18, 19). Comparing infection rates in canine’s population were 1% to 72.5% in the different countries (20, 21).

Interestingly in our research, *Spirometra* spp. was reported 2.38% and 6.25% in stray dogs and jackals, respectively. This parasite is intestinal parasite of canine and feline when these animals hunt intermediate or paratenic hosts such as amphibious, reptiles and small mammals (21, 22). Totally, prevalence of intestinal helminths in dog and jackals has shown significant difference in different areas of Mazandaran province and only infection of *A. caninum* showed a higher percentage in center region of province.

## Conclusion

There are still the clear risks of zoonotic helminths parasites infection in this region. Especially dogs are considered as an important reservoir for these helminthic infection diseases and it is dangerous for people in these areas. Therefore, understanding the epidemiology of zoonotic parasite infection is useful for health care access both domestic animals and humans health. Health education programs and control of the parasitic infections could be reduced the transmission of canine intestinal parasitic infections in the north of Iran.
